# Fiber Thickness and Porosity Control in a Biopolymer Scaffold 3D Printed through a Converted Commercial FDM Device

**DOI:** 10.3390/ma15072394

**Published:** 2022-03-24

**Authors:** Joseph Lovecchio, Marilisa Cortesi, Marco Zani, Marco Govoni, Dante Dallari, Emanuele Giordano

**Affiliations:** 1 Laboratory of Cellular and Molecular Engineering “Silvio Cavalcanti”, Department of Electrical, Electronic and Information Engineering “Guglielmo Marconi” (DEI), University of Bologna, 47521 Cesena, FC, Italy; marilisa.cortesi2@unibo.it (M.C.); emanuele.giordano@unibo.it (E.G.); 2 Gynaecological Cancer Research Group, Lowy Cancer Research Centre, Faculty of Medicine and Health, School of Women’s and Children’s Health, University of New South Wales, Sydney 2031, Australia; 3 Mark One S.r.l., 47521 Cesena, FC, Italy; zani.marco@3dmarkone.com; 4 Reconstructive Orthopaedic Surgery and Innovative Techniques-Musculoskeletal Tissue Bank, IRCCS Istituto Ortopedico Rizzoli, 40136 Bologna, RE, Italy; marco.govoni@ior.it (M.G.); dante.dallari@ior.it (D.D.); 5 BioEngLab, Health Science and Technology, Interdepartmental Center for Industrial Research (HST-CIRI), Alma Mater Studiorum, University of Bologna, 40064 Ozzano Emilia, BO, Italy; 6 Advanced Research Center on Electronic Systems (ARCES), University of Bologna, 40064 Ozzano Emilia, BO, Italy

**Keywords:** 3D bioprinting, FDM, bone tissue engineering, scaffolds, alginate, gelatin, fiber thickness, porosity, bone grafting

## Abstract

3D printing has opened exciting new opportunities for the in vitro fabrication of biocompatible hybrid pseudo-tissues. Technologies based on additive manufacturing herald a near future when patients will receive therapies delivering functional tissue substitutes for the repair of their musculoskeletal tissue defects. In particular, bone tissue engineering (BTE) might extensively benefit from such an approach. However, designing an optimal 3D scaffold with adequate stiffness and biodegradability properties also guaranteeing the correct cell adhesion, proliferation, and differentiation, is still a challenge. The aim of this work was the rewiring of a commercial fuse deposition modeling (FDM) 3D printer into a 3D bioplotter, aiming at obtaining scaffold fiber thickness and porosity control during its manufacturing. Although it is well-established that FDM is a fast and low-price technology, the high temperatures required for printing lead to limitations in the biomaterials that can be used. In our hands, modifying the printing head of the FDM device with a custom-made holder has allowed to print hydrogels commonly used for embedding living cells. The results highlight a good resolution, reproducibility and repeatability of alginate/gelatin scaffolds obtained via our custom 3D bioplotter prototype, showing a viable strategy to equip a small-medium laboratory with an instrument for manufacturing good-quality 3D scaffolds for cell culture and tissue engineering applications.

## 1. Introduction

Although conventional grafting techniques, involving the use of autografts and allogeneic or xenogeneic bone sources, are still widely used to treat bone defects [1], alternative strategies able to overcome the main pitfalls related to these current procedures, such as donor site morbidity, lack of tissue availability, infections, or low biocompatibility, are needed. Tissue engineering and regenerative medicine aim at developing functional tissue substitutes for repairing bone defects [2,3]. In recent decades, several studies focused on selecting cell sources, designing 3D scaffolds [4,5,6], developing bioreactors [7,8,9,10,11,12,13,14] to improve tissue commitment in vitro. However, designing an optimal 3D scaffold is still a challenge. Different biomaterials have been studied such as synthetic and natural polymers [15], which can also embed living cells inside [16], but several limitations are still present [2]. Indeed, each tissue has appropriate structural, mechanical, and biological properties promoting tissue formation, functioning, and remodeling. In this respect, the optimal bone tissue substitute should provide adequate stiffness and biodegradability to properly mimic the native extracellular matrix (ECM), and to guarantee the correct cell adhesion, proliferation, and differentiation [17]. To this aim, parameters such as 3D scaffold porosity and fiber thickness need to be properly controlled. This appears to be difficult with the conventional manual in vitro protocols. In this respect, 3D bioprinting techniques have currently started to be used [18]. The rationale comes from the development of conventional 3D printing, an additive manufacturing technology implemented in several fields, such as the research, automotive, aerospace, healthcare and medical contexts [19,20,21].

When tissue engineering settings are considered, 3D bioprinting allows using several biomaterials such as biopolymers, to manufacture tissue-like 3D micro- and macro-structures containing biochemicals and even living cells. Mastering this technology requires the setting of specific parameters for patterning biocompatible materials to manufacture 3D constructs with mechanical and biological properties suitable for the deposition of living cells. In this regard, laser-assisted [22,23], and extrusion [24] bioprinting afford good resolution, reproducibility and repeatability [25].

In this line of reasoning, the aim of this work was to explore whether a commercial fuse deposition modeling (FDM) 3D printer (ONE, Mark One, Italy; Available online: https://www.3dmarkone.com (accessed on 21 March 2022))— i.e., a non-biological 3D printer —could be converted into a 3D bioplotter with suitable fiber thickness and porosity control when deposing an alginate/gelatin model hydrogel solution. Indeed, FDM is a fast and low-price technology able to print a variety of materials. However, the high temperatures required for printing lead to limitations in using biomaterials, although recently more biocompatible materials have been adapted to FDM to create 3D scaffolds.

In this respect, modifying the printing head of the FDM device made it possible to convert it into a 3D bioplotter, and consequently to use cytocompatible model hydrogel formulation of potential use for future embedding of living cells.

## 2. Materials and Methods

### 2.1. Custom Readaptation of a FDM 3D Printer into a 3D Bioplotter

In a framework R&D partnership with the commercial manufacturer (Mark One, Mercato Saraceno, Italy), a FDM 3D printer was modified to develop a custom-made 3D bioprinter. To this aim, the 3D printer heating head was removed from the main body of the device and replaced with a custom-made holder. This maintains in position a 20G blunt-tip luer-lock needle used as biopolymer extruder. The needle was then connected, through a male/female luer-lock PVC tube ($20^{\prime \prime }$ HighPressure 900 PSI, Coleparmer, Bunker Ct Vernon Hills, IL, USA), to a 10 mL luer-lock syringe hosted in a motorized (NEMA 23—JK57HS56-2804, JKM, Nanjing, China) linear sliding guide (Jectsepc78tm2bw5, Jectse, Zhejiang, China).

### 2.2. Biopolymer Preparation

According to Gao et al. [26], an alginate/gelatin model hydrogel solution was prepared as a bioink to be extruded through the custom-made 3D bioplotter. Briefly, 0.04 g/mL (4% *w*/*v*) sodium alginate powder (A3249, Applichem, Darmstadt, Germany) and 0.05 g/mL (5% *w*/*v*) gelatin (type B—200 Bloom, Cameo, Italy) were added to deionized water and mixed through a magnetic stirrer (600 rpm, 60 °C, 30 min) to produce an alginate/gelatin solution. The bioink was then stored in 10 mL luer-lock syringes (i.e., bioink cartridges) at 4 °C.

### 2.3. Cross-Linker Preparation

To cross-link the alginate/gelatin bioink after its extrusion, Calcium Chloride (CaCl_2_) powder (C5080, Sigma Aldrich, Spruce St, Saint Louis, MO, USA) was added to deionized water and mixed through a magnetic stirrer (600 rpm, room temperature, 5 min) to produce a CaCl_2_ solution at a 40 mM concentration. The cross-linker solution was then stored in 50 mL falcon tubes at 4 °C.

### 2.4. 3D Scaffold Template Design

**Figure 1:**
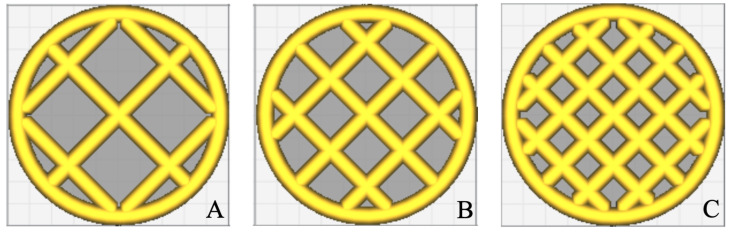
Circular shape with 35 (**A**), 50 (**B**), and 75 (**C**) % level of grid filling.

A series of 3D scaffold templates (Figure 1) were designed aiming at testing the custom-made 3D bioprinter. A circular shape 10 mm in diameter was obtained by the 123D Design CAD software (Autodesk, USA) and the file generated was exported in the .stl format. This file was imported in the Cura software (Ultimaker, Netherlands) and the circular shape was filled (Figure 1) with different levels of grid filling (i.e., 35, 50, and 75% of filler).

Subsequently, a print speed of 4 mm/s and an extrusion speed of 50 step/mm were set for the print process (Table 1). Each single .gcode file was exported on a mini-SD card, in order to be transferred to the custom-made 3D bioprinter.

### 2.5. 3D Bioprinter Setup

Before operating the printing, the syringe containing the bioink is heated up from 4 °C to 37 °C for 30 min. The syringe is then loaded on the motorized linear sliding guide and maintained at room temperature for 1 hour, to let the biopolymer reach its liquid phase and appropriate consistency. During this time, a Petri dish (60 × 15 mm, Orange Scientific, Braine-l’Alleud, Belgium) is washed with deionized water and placed on top of the print surface. A male/female luer-lock PVC tube is locked to the syringe on one end and to a 20G blunt-tip needle on the other. Finally, the distance between the needle and the Petri dish surface is set to 0.5 mm.

### 2.6. Data Collection

Six scaffolds for each level of grid filling were 3D bioprinted and immediately cross-linked with 1 mL 40 mM (CaCl_2_) solution for 1 min. 20 min after the end of the cross-linking procedure the diameter and height of each scaffold template were measured by a Vernier caliper with decimal readings. Pore size, pore area and fiber thickness of the grids, at 35, 50 and 75% of filler, were measured through a light microscope (Nikon Eclipse TE2000-U, Nikon, Tokyo, Japan) equipped with a A 5× objective (TU Plan Fluor 5X—NA 0.15, Nikon, Tokyo, Japan). Images were acquired via a DS-Qi1Mc camera (Nikon, Tokyo, Japan) controlled with a DS-U3 unit (Nikon, Tokyo, Japan). The NIS Elements software (AR 4.20.01, 64-bit, Nikon, Tokyo, Japan), was used for the quantification.

All the sizes were measured in three different positions for each scaffold template.

### 2.7. Statistical Analysis

The non-parametric Mann–Whitney–Wilcoxon test was implemented in the Matlab (Mathworks, Boston, MA, USA) environment to assess significant differences among groups. The significance level was set at *p* < 0.05. All results are presented as mean ± standard deviation.

## 3. Results

The 3D bioplotter detailed in this manuscript is a prototype from a commercial FDM device developed, within a framework R&D partnership with the commercial manufacturer (Mark One, Cesena, Italy), with the aim to print biocompatible polymers for tissue engineering purposes.

**Figure 2:**
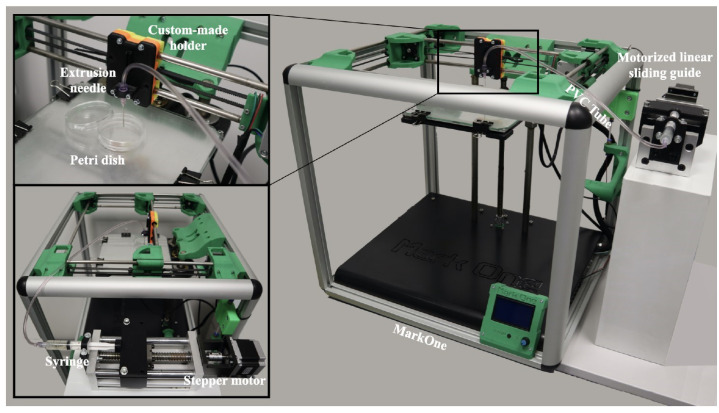
Final layout of the custom-made 3D bioplotter.

In its final layout, the 3D bioplotter (Figure 2) consists of an aluminum case of 50L × 50W × 60H cm in dimension, with a print volume of 20 cm × 20 cm × 30 cm in dimension extruded via a custom modified printhead. The biopolymer extrusion is actuated via a motorized linear sliding guide (26L × 11W × 8H cm in dimension) equipped with a NEMA 23 hybrid bipolar stepping motor delivering the extrusion speed of 50 step/mm. This linear sliding guide hosts the bioink cartridge of the printer. The cartridge is connected, via a male/female luer-lock PVC tube, to a 20G blunt-tip luer-lock needle, which is blocked on a custom-made holder replacing the original heating head of the FDM device. A Petri dish is placed on top of the printing surface at a distance of 0.5 mm from the needle, in order to guarantee the correct bioink extrusion.

### 3.1. Tuning 3D Bioplotter Operation Parameters

A series of trial-and-error tests were performed to estimate the correct parameters to be set for the 3D scaffold template printing. To this aim, the needle diameter, the distance between the needle and the Petri dish bottom surface, the print speed, and the extrusion speed were tested. Indeed, these parameters affect the accuracy of the 3D bioprinted construct, intended as the difference between the obtained and the expected object, and calculated through a measurement method [27] starting from the CAD model (reference standard) provided to the 3D printing device. To this aim, a simple test line was 3D printed using different blunt-tip needles with diameters in a range of 18–22G paired with different print (2–6 mm/s) and extrusion (50–150 step/mm) speeds. The most appropriate parameter combination resulted in the 20G blunt-tip luer-lock needle coupled with a print speed of 4 mm/s and an extrusion speed of 50 step/mm.

### 3.2. Influence of the Cross-Linking Solution on Biopolymer Reticulation

**Figure 3:**
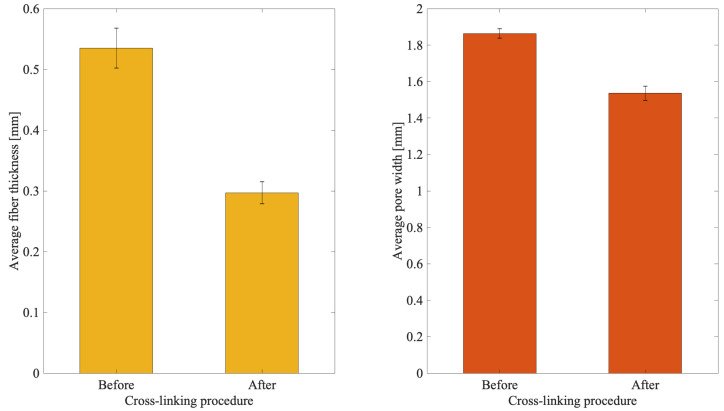
Influence of the cross-linking solution on the biopolymer. (**Left panel**), fiber thickness; (**Right panel**), pore width.

Measurements were performed to evaluate the effect of the cross-linking solution on the biopolymer reticulation. To this aim, n = 4 3D scaffold templates with a 50% filling grid were assessed before and after the use of a 40 mM (CaCl_2_) cross-linking solution. The fiber thickness and pore width were measured using a light microscope. Figure 3 shows the effect of the cross-linking solution over two measured parameters, i.e., fiber thickness and pore width.

A shrinkage of about 45% with respect to the original (i.e., before cross-linking) printed thickness was observed. Indeed, before biopolymer reticulation the fiber thickness was 535.75 ± 33.19 mm while immediately after it decreases to 297.05 ± 17.83 mm.

A similar but less pronounced behavior was observed for the pore width, where a shrinkage of about 18% of the printed pores was obtained. Indeed, before the biopolymer reticulation the pore width was 1864.16 ± 25.05 mm while immediately after it decreases to 1536.04 ± 39.04 mm.

### 3.3. 3D Scaffold Template Bioprinting

**Figure 4:**
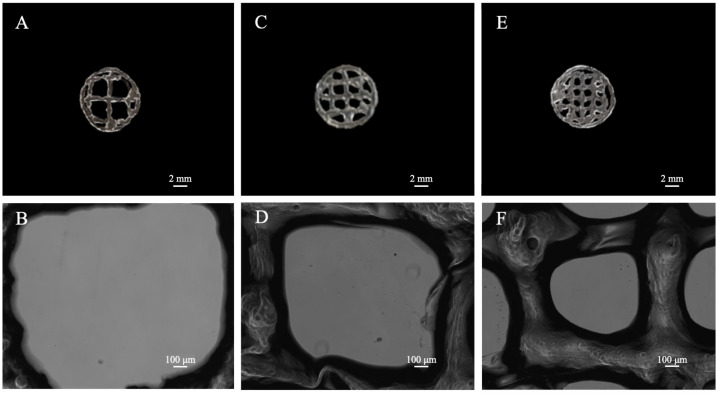
3D bioprinted scaffold template. Camera pictures ((**A**,**C**,**E**)—Scale bar 2 mm—Magnification 5×) and light microscope images ((**B**,**D**,**F**)—Scale bar 100 μm), respectively, for the circular shape with 35, 50, and 75% level of grid filling.

3D scaffold templates with different filling grids were 3D bioprinted and evaluated by a light microscope, in order to test the 3D bioprinter performance. To this aim, a circular shape of 10 mm in diameter was drawn by the 123D Design CAD software, imported in the Cura software and completed with different levels of grid filling (i.e., 35, 50, and 75% of filler; Figure 4). To set the levels, different line spacing were used as follows: 35% = line spacing 3.2 mm, 50% = line spacing 2.3 mm, 75% = line spacing 1.6 mm.

N = 6 samples were 3D-bioprinted for each 3D-scaffold template. Twenty minutes after the cross-linking procedure, each template was measured through a light microscope and n = 3 values were collected for the pore size, pore area and fiber thickness.

**Figure 5:**
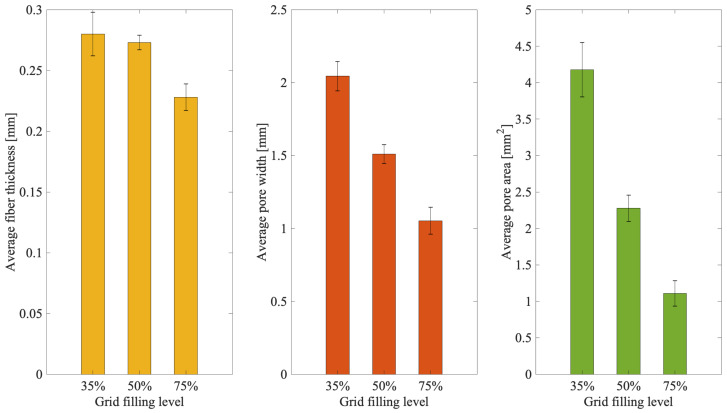
Evaluation of the 3D bioprinted scaffold templates with different grid fillings. (**Left panel**), fiber thickness; (**middle panel**), pore width; (**right panel**), pore area.

The microscope images show qualitatively as the pore size decreases consistently with the line spacing reduction. This is quantitatively confirmed by the performed measures (Figure 5). Indeed, decreasing values of pore width (about 2, 1.5, and 1 mm) and pore area (about 4, 2.25, and 1 mm^2^) were obtained, respectively, for templates at 35, 50, and 75% of grid filling.

**Figure 6:**
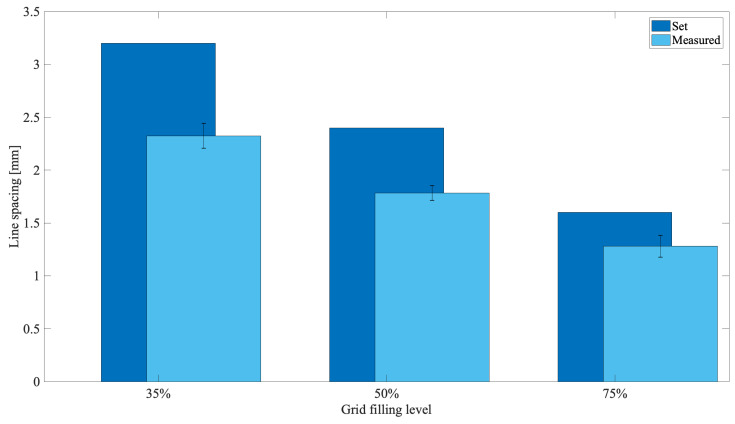
Evaluation of the 3D bioprinted scaffold templates, with different grid fillings, after the biopolymer reticulation.

When line spacing were measured, values of about 2.3, 1.8, 1.3 mm in comparison to those set of about 3.2, 2.4, 1.6 mm were scored (Figure 6), respectively, for the templates at 35, 50 and 75% of grid filling. An average shrinkage of about 25% was thus obtained, confirming the effect of the cross-linking solution on the biopolymer reticulation.

To define the highest resolution of the custom-made 3D bioprinter, lower line spacing values of 1.4, 1.2, 1 mm were tested. The lowest values of pore width (0.734 ± 0.024 mm) and pore area (0.512 ± 0.032 mm^2^) were obtained with 1.2 mm of line spacing, showing this as the highest reachable resolution. Indeed, below this threshold inhomogeneous templates and undetectable pores were obtained.

## 4. Discussion

Bioplotting techniques are currently in use for manufacturing bone tissue engineered 3D constructs with appropriate structural, mechanical, and biological properties [18]. However, the deposition processing of a biomaterial to obtain a biomimetic scaffold for living cells growing onboard is still a challenge. In fact, parameters such as scaffold porosity and fiber thickness need a tight control [2,16,17]. Commercial bioplotters are expensive devices requiring specific competences to manufacture scaffolds with good resolution, reproducibility, and repeatability. The budget to acquire such instruments represents a significant burden on a small-medium tissue engineering (TE) laboratory. Thus, the aim of this work was to explore if a commercial FDM 3D printer (M1, MarkOne, Mercato Saraceno, Italy; Available online: https://www.3dmarkone.com (accessed on 21 March 2022))—i.e., a non-biological 3D printer—could be converted into a 3D bioplotter, gaining suitable fiber thickness and porosity control during the 3D scaffold manufacture. FDM is a relatively low-cost technology able to print a variety of materials supplied as fibers on a reel that need to be locally fused by a printhead [28]. However, the high temperatures required for printing lead to limitations in the biomaterials that can be used, not to mention their killing effects on potentially embedded living cells. In this respect, the printing head of our prototypal device was modified within a framework R&D partnership with the manufacturer (Mark One, Cesena, Italy), to allow the deposition of a alginate/gelatin model hydrogel using a 20G blunt-tip luer-lock needle, which was blocked on a custom-made holder replacing the original heating head of the FDM device (Figure 2). The needle was connected via a male/female luer-lock PVC tube to a bioink cartridge hosted within a motorized linear sliding guide allowing biopolymer extrusion (Figure 2). The correct parameters (i.e., needle diameter, print and extrusion speeds) for the 3D scaffold template printing were set through a series of trial-and-error tests in order to optimize the accuracy of the 3D bioprinted construct (data not shown). The needle diameter proved to play an important role during biopolymer extrusion: needles with a diameter smaller than 20G did not allow the biopolymer extrusion due to needle occlusion; on the opposite, needles with a diameter bigger than 20G lead to inaccurately extruded biopolymer templates. Thus, the larger is the needle diameter, the lower will be the resolution and the accuracy of the 3D printed scaffold template (data not shown). Accuracy control also involves the coherence of the actual 3D printed scaffold dimensions with those originally designed as per project. In fact, the biopolymer construct needs to be reticulated upon extrusion, using a CaCl_2_ crosslinking solution to induce an adequate mechanical stability. This procedure affected the printed fiber thickness that shrunk to about 55% of the originally planned dimension (Figure 3, left panel). This process is finally reflected over the overall scaffold size, thus over the scaffold’s pore width that shrunk to 82% of the pre-crosslinking procedure (Figure 3, right panel). Since this step is mandatory to guarantee the stability of the 3D bioprinted scaffold templates, such a variation in size of these structures must always be taken into account and the original design dimensions need to be increased accordingly. Such a coherence can be obtained via the tuning of print and extrusion speed control parameters. Here this was obtained by a trial-and-error approach setting the desired values using the Cura open source software, allowing translating the original .stl CAD file into G-code, the machine language that commands the printer. The prototype was therefore tested printing templates with a circular shape and different filling grids (Figure 1 and Figure 4), i.e., 35% = line spacing 3.2 mm, 50% = line spacing 2.3 mm, 75% = line spacing 1.6 mm. After the cross-linking procedure, an average shrinkage of about 25% was obtained for the fiber thickness, pore size, and pore area (Figure 5) of all templates, as an expected effect of the cross-linking solution on the biopolymer reticulation. These results, showing narrow standard deviation values for each measured parameter (Figure 6) highlight a good resolution, reproducibility and repeatability of scaffolds obtained via our custom 3D bioplotter prototype. To detect the highest resolution of the 3D bioplotter, lower line spacing values of 1.4, 1.2, 1 mm were tested. In particular, grids with a line spacing lower than 1.2 mm showed (data not shown) that this is the highest resolution allowed. In fact, inhomogeneous templates with undetectable pores were obtained below this threshold. To check for bioink cartridge stability, our tests were carried out 1 and 7 days after hydrogel alginate/gelatin mix was prepared. No statistically significant differences were scored among the two time points, thus obtained data were pooled in Figure 6, where the narrow SD values credit the restricted dispersion of measures. This is considered an additional advantage given that one can rely on a significant time to use each bioink batch.

Taken as a whole, this work of ours is part of a series of recent research contributions [29,30,31,32,33,34] aiming at the ease of use and cost-effectiveness of a 3D bioprinting process, to be offered to life sciences researchers for a wide range of applications in tissue engineering and regenerative medicine protocols [35].

Converting a commercial FDM 3D printer into a bioplotter, aiming at obtaining fiber thickness and porosity control during the 3D scaffold manufacture, appears to be a strategy to equip a small-medium laboratory with a device for manufacturing 3D scaffolds suitable for cell culture.

## Figures and Tables

**Table 1:** Printing parameters.

Parameter	Value
Bioink cartridge	Sodium alginate (4% *w*/*v*) Gelatin (5% *w*/*v*)
CaCl_2_	40 mM
Print speed	4 mm/s
Extrusion speed	50 step/mm

## Data Availability

Not applicable.

## References

[1] Govoni M., Vivarelli L., Mazzotta A., Stagni C., Maso A., Dallari D. (2021). Commercial Bone Grafts Claimed as an Alternative to Autografts: Current Trends for Clinical Applications in Orthopaedics. Materials.

[2] Willerth S.M., Sakiyama-Elbert S.E. (2019). Combining Stem Cells and Biomaterial Scaffolds for Constructing Tissues and Cell Delivery. StemJournal.

[3] Perić Kačarević Ž., Rider P., Alkildani S., Retnasingh S., Pejakić M., Schnettler R., Gosau M., Smeets R., Jung O., Barbeck M. (2020). An introduction to bone tissue engineering. Int. J. Artif. Organs..

[4] Pati F., Ha D.-H., Jang J., Han H.H., Rhie J.-W., Cho D.-W. (2015). Biomimetic 3D Tissue Printing for Soft Tissue Regeneration. Biomaterials.

[5] Choi Y.-J., Kim T.G., Jeong J., Yi H.-G., Park J.W., Hwang W., Cho D.-W. (2016). 3D Cell Printing of Functional Skeletal Muscle Constructs Using Skeletal Muscle-Derived Bioink. Adv. Healthc. Mater..

[6] Picone G., Cappadone C., Pasini A., Lovecchio J., Cortesi M., Farruggia G., Lombardo M., Gianoncelli A., Mancini L., Ralf H.M. (2020). Analysis of Intracellular Magnesium and Mineral Depositions during Osteogenic Commitment of 3D Cultured Saos2 Cells. Int. J. Mol. Sci..

[7] Lovecchio J., Jónsdóttir-Buch S.M., Einarsdóttir G.K., Gíslason M.K., Örlygsson G., Sigurjónsson Ó.E., Gargiulo P. (2014). Assessment of a Perfusion Bioreactors System using *μ*CT Technology and 3D Modeling Methods. Biomed. Eng./Biomed. Technik.

[8] Govoni M., Muscari C., Lovecchio J., Guarnieri C., Giordano E. (2015). Mechanical Actuation Systems for the Phenotype Commitment of Stem Cell-Based Tendon and Ligament Tissue Substitutes. Stem Cell Rev. Rep..

[9] Pasini A., Lovecchio J., Ferretti G., Giordano E. (2019). Medium Perfusion Flow Improves Osteogenic Commitment of Human Stromal Cells. Stem Cells Int..

[10] Lovecchio J., Pannella M., Giardino L., Calzà L., Giordano E. (2019). A Dynamic Culture Platform Enhances the Efficiency of the 3D HUVEC-based Tube Formation Assay. Biotechnol. Bioeng..

[11] Lovecchio J., Gargiulo P., Vargas Luna J.L., Giordano E., Sigurjónsson Ó.E. (2019). A Standalone Bioreactor System to Deliver Compressive Load under Perfusion Flow to HBMSC-Seeded 3D Chitosan-Graphene Templates. Sci. Rep..

[12] Ciardulli M.C., Marino L., Lovecchio J., Giordano E., Forsyth N.R., Selleri C., Maffulli N., Porta G.D. (2020). Tendon and Cytokine Marker Expression by Human Bone Marrow Mesenchymal Stem Cells in a Hyaluronate/Poly-Lactic-Co-Glycolic Acid (PLGA)/Fibrin Three-Dimensional (3D) Scaffold. Cells.

[13] Pasini A., Lovecchio J., Cortesi M., Liverani C., Spadazzi C., Mercatali L., Ibrahim T., Giordano E. (2021). Perfusion Flow Enhances Viability and Migratory Phenotype in 3D-Cultured Breast Cancer Cells. Ann. Biomed. Eng..

[14] Lamparelli E.P., Lovecchio J., Ciardulli M.C., Giudice V., Dale T.P., Selleri C., Forsyth N., Giordano E., Maffulli N., Della Porta G. (2021). Chondrogenic Commitment of Human Bone Marrow Mesenchymal Stem Cells in a Perfused Collagen Hydrogel Functionalized with HTGF-*β*1-Releasing PLGA Microcarrier. Pharmaceutics.

[15] Reddy M.S.B., Ponnamma D., Choudhary R., Sadasivuni K.K. (2021). A Comparative Review of Natural and Synthetic Biopolymer Composite Scaffolds. Polymers.

[16] Rodrigo-Navarro A., Sankaran S., Dalby M.J., del Campo A., Salmeron-Sanchez M. (2021). Engineered Living Biomaterials. Nat. Rev. Mater..

[17] Fergal J.O. (2011). Biomaterials & scaffolds for tissue engineering. Mater. Today.

[18] Genova T., Roato I., Carossa M., Motta C., Cavagnetto D., Mussano F. (2020). Advances on Bone Substitutes through 3D Bioprinting. Int. J. Mol. Sci..

[19] Obolid I.T., Peng W., Ozbolat V. (2016). Application areas of 3D bioprinting. Drug Discov. Today.

[20] Khorasani M., Ghasemi A., Rolfe B., Gibson I. (2022). Additive manufacturing a powerful tool for the aerospace industry. Rapid Prototyp. J..

[21] Tan C., Zou J., Li S., Jamshidi P., Abena A., Forsey A., Moat R.J., Essa K., Wang M., Zhou K. (2021). Additive manufacturing of bio-inspired multi-scale hierarchically strengthened lattice structures. Int. J. Mach. Tools Manuf..

[22] Saini G., Segaran N., Mayer J.L., Saini A., Albadawi H., Oklu R. (2021). Applications of 3D Bioprinting in Tissue Engineering and Regenerative Medicine. J. Clin. Med..

[23] Koch L., Brandt O., Deiwick A., Chichkov B. (2017). Laser-assisted bioprinting at different wavelengths and pulse durations with a metal dynamic release layer: A parametric study. Int. J. Bioprint.

[24] Chang C.C., Boland E.D., Williams S.K., Hoying J.B. (2011). Direct-write bioprinting three-dimensional biohybrid systems for future regenerative therapies. J. Biomed. Mater. Res. B Appl. Biomater..

[25] Singh M., Haverinen H.M., Dhagat P., Jabbour G.E. (2010). Inkjet printing-process and its applications. Adv. Mater..

[26] Gao T., Gillispie G.J., Copus J.S., Pr A.K., Seol Y.-J., Atala A., Yoo J.J., Lee S.J. (2018). Optimization of gelatin–alginate composite bioink printability using rheological parameters: A systematic approach. Biofabrication.

[27] Alruwaili M., Lopez J.A., McCarthy K., Reynaud E.G., Rodriguez B.J. (2019). Liquid-Phase 3D Bioprinting of Gelatin Alginate Hydrogels: Influence of Printing Parameters on Hydrogel Line Width and Layer Height. Bio-Des. Manuf..

[28] Chung J.J., Im H., Kim S.H., Park J.W., Jung Y. (2020). Toward Biomimetic Scaffolds for Tissue Engineering: 3D Printing Techniques in Regenerative Medicine. Front. Bioeng. Biotechnol..

[29] Kahl M., Gertig M., Hoyer P., Friedrich O., Gilbert D.F. (2019). Ultra-Low-Cost 3D Bioprinting: Modification and Application of an Off-the-Shelf Desktop 3D-Printer for Biofabrication. Front. Bioeng. Biotechnol..

[30] Raza M.H., Abdullah M., Rehman M.U., Mubarak Z., Arshad M. Development of An Extrusion 3D Bioprinter for Bioprinting of Hydrogel Based Biomaterials. Proceedings of the International Conference on Robotics and Automation in Industry (ICRAI).

[31] Bociaga D., Bartniak M., Sobczak K., Rosinska K. (2020). An Integration of a Peristaltic Pump-Based Extruder into a 3D Bioprinter Dedicated to Hydrogels. Materials.

[32] Ioannidis K., Danalatos R.I., Champeris Tsaniras S., Kaplani K., Lokka G., Kanellou A., Papachristou D.J., Bokias G., Lygerou Z., Taraviras S. (2020). A Custom Ultra-Low-Cost 3D Bioprinter Supports Cell Growth and Differentiation. Front. Bioeng. Biotechnol..

[33] Koch F., Thaden O., Tröndle K., Zengerle R., Zimmermann S., Koltay P. (2021). Open-source hybrid 3D-bioprinter for simultaneous printing of thermoplastics and hydrogels. HardwareX.

[34] Wagner M., Karner A., Gattringer P., Buchegger B., Hochreiner A. (2021). A super low-cost bioprinter based on DVD-drive components and a raspberry pi as controller. Bioprinting.

[35] Xie Z., Gao M., Lobo A.O., Webster T.J. (2020). 3D Bioprinting in Tissue Engineering for Medical Applications: The Classic and the Hybrid. Polymers.

